# Actinomycetoma of the wrist: diagnostic insights from a pathology perspective (case report)

**DOI:** 10.11604/pamj.2026.53.125.48522

**Published:** 2026-03-12

**Authors:** Aaliya Sheikh, Keshao Hiwale, Shailly Tiwari

**Affiliations:** 1 Department of Pathology, Datta Meghe Institute of Medical Science and Research, Sawangi Meghe, Wardha, Maharashtra, India

**Keywords:** Actinomycetoma, wrist, *Nocardia brasiliensis*, sulphur grains, case report

## Abstract

Actinomycetoma is a chronic, progressive subcutaneous infection caused by filamentous bacteria of the Actinomycetales order, characterized by tumefaction, sinuses, and grains. We report a rare case of actinomycetoma affecting the wrist of a 45-year-old male, which presented as a chronic, slowly enlarging swelling with multiple discharging sinuses. Initial clinical suspicion was broad, encompassing various chronic infections or soft tissue tumors. However, definitive diagnosis was established through meticulous histopathological examination of a biopsy specimen, revealing characteristic "sulphur grains" composed of a basophilic bacterial colony surrounded by Splendore-Hoeppli phenomenon, alongside a mixed inflammatory infiltrate. Culture subsequently confirmed Nocardia brasiliensis. This case underscores the crucial role of integrated histopathology and microbiology in distinguishing actinomycetoma from other chronic granulomatous conditions, particularly in atypical anatomical locations, ensuring timely and appropriate therapeutic intervention.

## Introduction

Actinomycetoma is a localized, chronic subcutaneous infection caused by specific aerobic actinomycetes, most commonly *Nocardia, Actinomadura*, and *Streptomyces* species [1]. It typically presents with a triad of tumefaction (swelling), multiple discharging sinuses, and the extrusion of characteristic 'grains' (microcolonies of the causative organism) [2]. While endemic in tropical and subtropical regions [3], its occurrence on the wrist is relatively uncommon compared to the feet [4,5]. Diagnostic challenges often arise due to its slow progression, diverse clinical presentations, and mimicry of other chronic granulomatous diseases or neoplastic processes [2,6]. This case highlights the indispensable role of pathology in confirming the diagnosis, especially when clinical features are non-specific or the anatomical site is unusual.

## Patient and observation

**Patient information:** a 45-year-old male farmer, residing in an endemic rural area, presented with a 1-year history of a slowly progressive, painless swelling on his right wrist. The swelling had gradually increased in size and was associated with multiple raised nodules and intermittently discharging sinuses, exuding a seropurulent discharge. He denied any history of recent trauma, fever, or constitutional symptoms. His past medical history was unremarkable.

**Figure 1:**
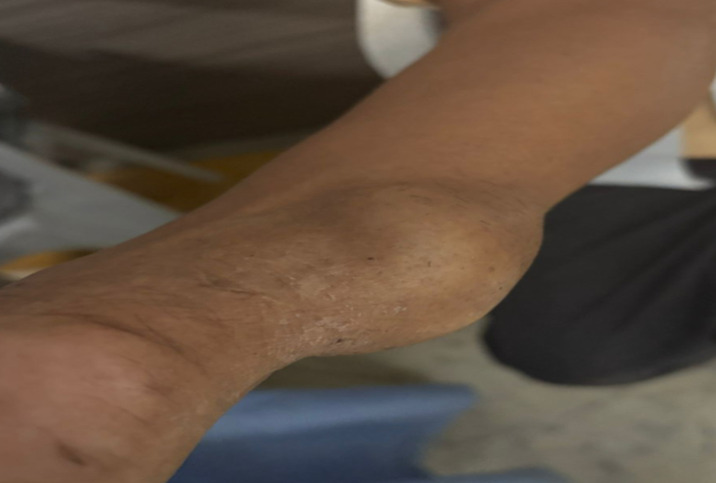
dorsal aspect of the right wrist showing chronic swelling, multiple nodules, and discharging sinus tracts

**Clinical findings:** on physical examination, a firm, non-tender, indurated swelling measuring approximately 5x4 cm was noted on the dorsal aspect of the right wrist, extending towards the carpal region. Overlying skin showed areas of hyperpigmentation, scarring, and multiple actively discharging sinuses. No regional lymphadenopathy was observed. The patient had restricted wrist movement due to the swelling and induration (Figure 1).

**Timeline of the current episode:** the patient presented with a 1-year history of a slowly progressive, painless swelling on his right wrist, gradually increasing in size and associated with multiple nodules and intermittently discharging sinuses.

### Diagnostic assessment

#### Imaging

**X-ray of the right wrist:** revealed soft tissue swelling and underlying subtle periosteal reaction involving the distal radius and carpal bones, without overt osteolytic lesions, suggesting chronic inflammatory changes rather than aggressive malignancy.

**Magnetic resonance imaging (MRI) of the right wrist:** demonstrated extensive involvement of subcutaneous tissues, muscle, and periosteum, with multiple small fluid collections and sinus tracts. Signal characteristics were consistent with a chronic inflammatory process. While supportive of infection, imaging alone could not definitively differentiate it from other chronic granulomatous diseases or some slow-growing tumors.

**Laboratory tests:** routine blood counts were within normal limits, with a mild elevation in erythrocyte sedimentation rate (ESR) at 35 mm/hr (normal <20 mm/hr). C-reactive protein (CRP) was also mildly elevated at 12 mg/L (normal <5 mg/L).

### Pathological and microbiological investigations (central to diagnosis)

**Direct microscopic examination of discharge:** a sample of the discharge from an active sinus tract was examined under a microscope. Wet mount preparation revealed small, irregularly shaped, yellowish-white "grains." When crushed and stained with Gram stain, these grains showed dense aggregates of fine, branching, Gram-positive filamentous organisms.

**Biopsy specimen:** an incisional biopsy of the wrist lesion was performed, including skin, subcutaneous tissue, and deeper muscle.

**Figure 2:**
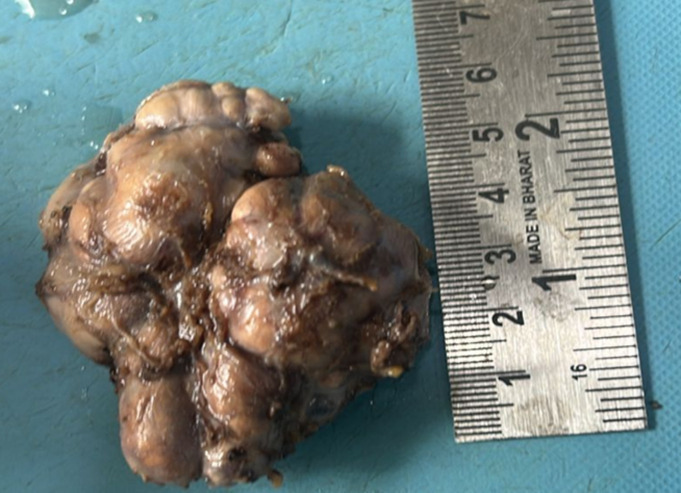
gross specimen: excised specimen showing multilobulated mass

**Figure 3:**
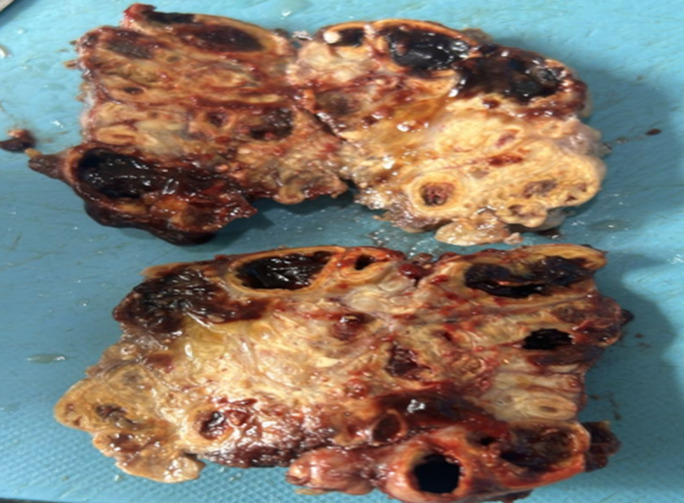
gross specimen: on cut section of excised specimen showing indurated tissue with visible yellowish-white 'grains' and multiple cystic and solid areas

**Gross pathology:** the excised specimen consisted of a multilobulated grey-white tissue mass measuring approximately 7 x 5.5 x 2.3 cm (Figure 2). On cut section, multiple cystic and solid areas were seen and several small, yellowish-white, firm 'grains' were grossly visible (Figure 3).

**Figure 4:**
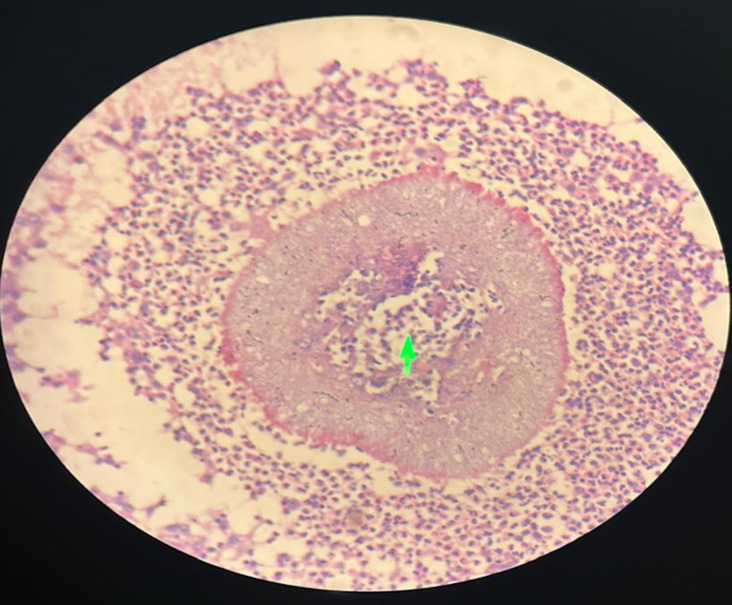
histopathology (H&E stain, high power): magnified view of a "sulphur grain" showing a central basophilic bacterial colony surrounded by the eosinophilic Splendore-Hoeppli phenomenon

**Figure 5:**
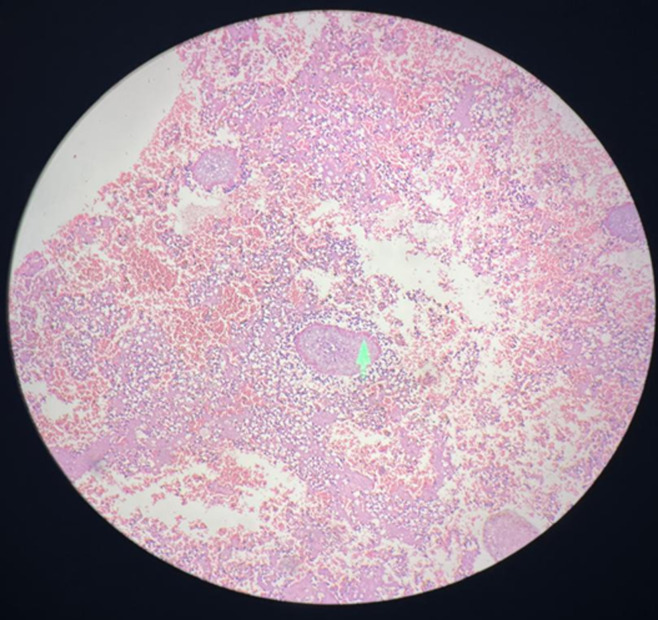
histopathology (H&E stain, low power): section demonstrating a "sulphur grain" within a dense inflammatory infiltrate and surrounding fibrous tissue

**Figure 6:**
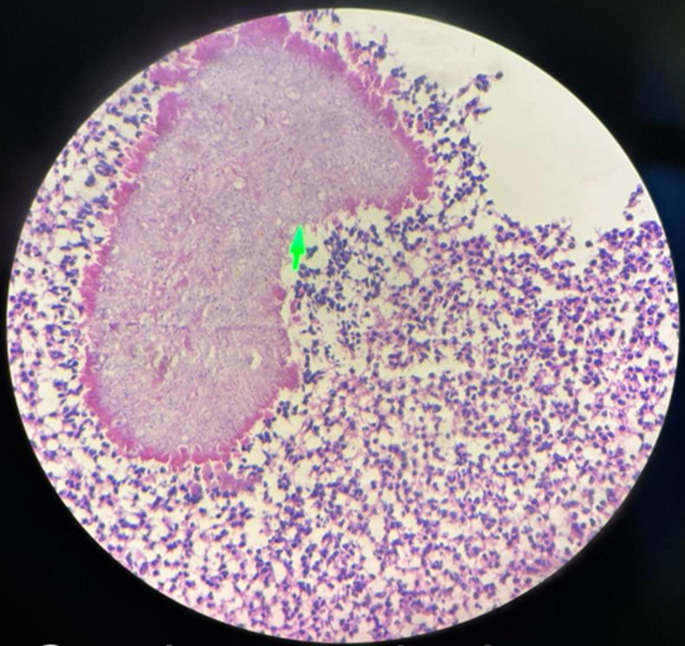
histopathology (H&E stain, high power): section demonstrating a sun burst appearance within a dense inflammatory infiltrate

**Histopathological examination (H&E stain):** sections showed characteristic "sulphur grains" embedded within a polymorphous inflammatory infiltrate. Each grain was composed of a central, densely packed, basophilic colony of filamentous microorganisms. The periphery of these colonies exhibited the Splendore-Hoeppli phenomenon, appearing as radiating eosinophilic club-like structures due to the deposition of host antigen-antibody complexes (Figure 4, Figure 5). The surrounding inflammatory response was chronic and granulomatous, consisting of neutrophils, macrophages, lymphocytes, plasma cells, and occasional giant cells. Extensive fibrosis and multiple sinus tracts lined by granulation tissue were also noted, consistent with chronic infection and presence of classical sun burst appearance within a dense inflammatory infiltrate (Figure 6).

### Special stains

**Gram stain:** applied to tissue sections, confirmed the Gram-positive nature of the filamentous organisms within the grains.

**Modified Ziehl-Neelsen (ZN) stain:** performed to assess for partial acid-fastness. The filamentous organisms within the grains showed partial acid-fastness, strongly suggesting *Nocardia* species [7].

**Periodic Acid-Schiff (PAS) and Grocott's Methenamine Silver (GMS) stains:** these stains were negative for fungal elements, effectively ruling out eumycetoma.

### Microbiological culture

Tissue and discharge samples were inoculated onto various media including Sabouraud Dextrose Agar (SDA) (with and without antibiotics), blood agar, and Löwenstein-Jensen (LJ) medium; aerobic incubation at 37°C yielded slow-growing, chalky-white, wrinkled colonies on blood agar after 7-10 days; biochemical tests and subsequent 16S rRNA gene sequencing identified the isolate as *Nocardia brasiliensis*.

**Differential diagnosis:** the clinical presentation of a chronic, indurated swelling with discharging sinuses on the wrist necessitated a comprehensive differential diagnosis, which was primarily elucidated by detailed pathological and microbiological investigations.

**Eumycetoma:** clinically similar, but caused by true fungi. This was definitively ruled out by the absence of fungal elements on PAS and GMS stains, and the identification of bacterial filaments and their partial acid-fastness on modified ZN stain.

**Chronic osteomyelitis/tuberculosis:** these can present with chronic inflammation, bone involvement, and sinus tracts. While imaging showed periosteal reaction, the presence of specific 'grains' and the identification of *Nocardia* on microscopy and culture differentiated actinomycetoma. Ziehl-Neelsen staining for acid-fast bacilli was negative.

**Atypical mycobacterial infections:** these can cause chronic skin and soft tissue infections. While *Nocardia* are partially acid-fast, molecular identification and specific culture characteristics helped distinguish them from atypical mycobacteria.

**Deep fungal infections (e.g. *sporotrichosis, chromoblastomycosis*):** these can mimic actinomycetoma, especially in terms of subcutaneous nodules and ulcerations. However, specific fungal morphology and culture characteristics were absent.

**Soft tissue tumors (benign/malignant):** slowly growing tumors could be considered clinically. However, the presence of active discharging sinuses and characteristic inflammatory infiltrates with grains on biopsy ruled out a primary neoplastic process.

**Foreign body granuloma:** possible with a history of trauma, but the presence of a specific microbial etiology on microscopy and culture excluded this.

The crucial differentiating factor in this case was the histopathological identification of the "sulphur grains" with their specific bacterial morphology, confirmed by special stains and ultimately the definitive culture and molecular identification of *Nocardia brasiliensis*.

**Therapeutic interventions:** based on the diagnosis of *Nocardia brasiliensis* actinomycetoma, the patient was initiated on a long-term combination antibiotic regimen consisting of co-trimoxazole (trimethoprim/sulfamethoxazole) and amikacin. The treatment duration was planned for 6-12 months, contingent on clinical response and resolution of lesions. Surgical debridement was considered but not immediately performed due to the extent of the lesion and good response to initial medical therapy.

**Follow-up and outcome:** the patient showed significant clinical improvement within 2-3 months of starting antibiotic therapy. The swelling on the wrist gradually subsided, and the discharging sinuses healed. Wrist mobility improved. He completed a 9-month course of antibiotics. At a 12-month follow-up, the lesion had completely resolved with residual scarring and full restoration of wrist function. There were no signs of recurrence.

**Patient perspective:** "living with a swelling on my wrist that kept discharging and making my hand stiff was very frustrating and painful. As a farmer, my hands are crucial for my work, and this made daily tasks incredibly difficult. I worried constantly about what it could be and if I would ever regain full use of my hand. When the doctors finally told me what it was and that it could be treated with medicine, I felt a huge sense of relief. The long course of treatment was challenging, but seeing the swelling go down and the sinuses heal, and being able to use my wrist normally again, has been truly life-changing. I am very thankful to the medical team for their thorough diagnosis and effective treatment."

**Informed consent:** written informed consent was obtained from the patient for publication of this case report and accompanying images.

## Discussion

This case of actinomycetoma affecting the wrist underscores several important points in the diagnosis and management of this challenging chronic infection. Actinomycetoma, though primarily affecting the foot, can occur at any anatomical site, including the upper extremities, making clinical diagnosis difficult, especially in the absence of typical ''sulphur grains'' in the discharge [4,5]. The wrist, being a weight-bearing and frequently exposed area in farmers, can be a site of inoculation. The pivotal role of pathology in this case cannot be overstated. The initial clinical presentation was non-specific, leading to a broad differential diagnosis. Imaging provided anatomical detail but lacked specificity. The presence of ''sulphur grains'' in the discharge, followed by the classic histopathological findings of basophilic filamentous bacterial colonies (grains) surrounded by the Splendore-Hoeppli phenomenon and a chronic granulomatous inflammatory response, was pathognomonic [2]. The partial acid-fastness of the organisms on modified ZN stain narrowed the etiology to Nocardia, which was subsequently confirmed by culture and molecular identification [7]. This sequential diagnostic approach, integrating clinical suspicion, imaging, direct microscopy, histopathology, special stains, and culture, is essential for accurate diagnosis of mycetoma (both actinomycetoma and eumycetoma) [6]. Early and accurate diagnosis prevents prolonged suffering, extensive tissue destruction, and inappropriate treatment, as misdiagnosis can lead to unnecessary surgical interventions or ineffective antibiotic regimens. The successful outcome with long-term antibiotic therapy highlights the curability of actinomycetoma when diagnosed early and treated appropriately [8].

## Conclusion

This case emphasizes several key learning points for the diagnosis and management of actinomycetoma. Actinomycetoma should always be considered in the differential diagnosis of chronic, slowly progressive subcutaneous swellings with discharging sinuses, even when they appear in atypical anatomical locations such as the wrist [9]. Histopathological identification of the characteristic "sulphur grains," which are composed of filamentous bacteria surrounded by the Splendore-Hoeppli phenomenon, is pathognomonic for this condition [10]. Furthermore, the use of special stains (Gram, modified Ziehl-Neelsen, PAS, GMS) is crucial for differentiating actinomycetoma from eumycetoma and other granulomatous conditions. Ultimately, microbiological culture and molecular techniques are essential for definitive species identification, such as *Nocardia brasiliensis*, which directly guides specific long-term antibiotic therapy. An early and accurate pathological diagnosis is critical for effective management, preventing extensive tissue destruction, and reducing morbidity in patients with actinomycetoma.
